# Monitoring of the auditory pathway maturation after early intervention during the first year of life in infants with sensorineural hearing loss

**DOI:** 10.1007/s00405-020-06498-3

**Published:** 2020-12-18

**Authors:** F. Matin, S. Haumann, W. Roßberg, D. Mitovska, T. Lenarz, A. Lesinski-Schiedat

**Affiliations:** Otorhinolaryngology Department, Head and Neck Surgery, Hanover Medical University, Hannover, Germany

**Keywords:** Auditory pathway maturation, Hearing aid, Infant, Auditory brainstem response, Follow-up, Hearing loss

## Abstract

**Purpose:**

The objective of this study was to investigate the auditory pathway maturation monitored by auditory brainstem responses (ABR) in infants with hearing loss during the first year of life. ABR were used to estimate hearing thresholds and the effect of early intervention strategies using hearing aids (HA).

**Methods:**

Click-evoked ABRs were measured in 102 infants aged from 0 to 12 months to determine their individual auditory threshold. Early therapy intervention was recommended before 12 months of age and analyzed. To evaluate the effect of hearing amplification on auditory maturation, different subgroups of infants with moderate hearing loss were analyzed and the auditory pathway maturation was determined based on IPL I–V shortening.

**Results:**

Overall, 110 ears (54.0% of 204 ears) with mild to profound HL showed threshold changes of 10 dB up to 60 dB in the follow-up ABR testing. HA were prescribed at the age of 3.8 ± 3.9 months. Cochlear implantation (CI) was performed in cases of repeated profound HL at the age of 9.9 months ± 4.5 months. A significant shortening of IPL I–V in all subgroups of infants (with and without risk factors) who received HA was shown and assumed auditory pathway maturation.

**Conclusion:**

An early intervention using optimally fitted HA influenced auditory pathway maturation and may lead to improvements of hearing thresholds during the first year of life in infants. This study underscores the importance of not only providing HAs to infants, but also controlling for hearing threshold changes ensuring that HAs provide the optimal level of intervention or CI is indicated.

## Introduction

Severe to profound bilateral sensorineural hearing loss (SNHL) is present in 1 to 3 out of 1000 newborns and 2 to 4 out of 100 infants who require neonatal intensive care (American Academy of Pediatrics 1999) [1–3]. To avoid significant delay of speech and language acquisition as well as auditory maturation followed by undetected congenital hearing loss (HL), an early identification and intervention is required [4]. After establishing the universal newborn hearing screening (UNHS) worldwide, the early detection of HL in infants has improved [5, 6]. In case of repeated negative hearing screening, diagnostic audiometry is necessary to determine hearing threshold. Auditory brainstem response (ABR) constitutes one of the most reliable responses of function of the auditory nerve and auditory brainstem pathways [7, 8]. The test can be performed during postprandial sleep, under sedation or general anesthesia. Previous studies revealed that ABR measurement is a very reliable tool in diagnosing hearing impairment and maturational changes of the auditory system from the periphery to the brainstem level [9, 10]. In ABR wave, Jewett V thresholds for evaluating the degree of HL and the interpeak latency (IPL) of wave Jewett I–V for identifying auditory maturation are usually measured [11].

The ability to obtain reliable behavioral responses in infants is useful clinically [12]. Even though capacities of behavioral audiometry are recognized in infants from 5 to 6 months onwards, this method of exploring hearing is often considered to be unfeasible or unreliable below this age [12–15]**.** The click-evoked ABR provides reliable estimates of the behavioral pure-tone thresholds in the frequency range 2–4 kilohertz (kHz). Using special fitting and stimulus configurations, more frequency-specific responses down to 1 kHz can be evoked [11, 16].

The central auditory system is immature at birth. The main neuronal maturation occurs within the first 2 years of life [8, 11, 17]. Even more importantly, the decisive part of the auditory maturation with the main shortening of the IPLs of the auditory evoked potentials (AEPs) takes place within the first year of life in infants [18].

Certain risk factors have been associated with HL in children [19, 20] and the risk of delayed maturation of auditory pathway**.** These risk factors include family history of HL, in utero infections, craniofacial anomalies, diagnosis of a HL-associated syndrome, postnatal infections and head traumata [21]. Perinatal causes of HL include prematurity, prolonged neonatal intensive care, hypoxia, low Apgar scores and hyperbilirubinemia, all of these can cause selective damage to the brainstem auditory nuclei and may damage the auditory nerve and ganglion cells [22]. Severe hypoxia may cause irreversible cellular damage to the stria vascularis in the cochlea and the outer hair cells [23]. All infants identified with a risk factor for developing HL should receive appropriate early diagnostic procedures to avoid a delay between diagnosis and intervention.

The possibility of hearing threshold changes in infants as described in numerous previous studies on follow-up ABR measurements draws attention to the need of clear discrimination of the HL type at the time of diagnostic testing as well as the performance of follow-up ABR testing of diagnosed SNHL [24]. In a study conducted by Kang et al. of 71 infants with SNHL, follow-up ABR testing showed in 34 percent a hearing threshold improvement of more than 20 decibel (dB) where even 30 percent of these recovered to normal hearing. Out of the same population, 8 percent showed a deterioration of more than 20 dB [5]. The study of Lim et al. also reported improvement in hearing in infants with confirmed HL. Three (13%) of the 23 subjects showed changes in severity of HL at the follow up ABR testing [24]. In another study of Talero-Gutiérrez et al. [25,] the ABR of 25 infants improved in morphology and response thresholds in 32 percent. The developmental changes observed included a synchrony in the wave morphology, decreased latencies of the late waves (III and V) and decreased IPLs [25]. But when threshold deterioration is confirmed in the follow-up ABR measurements, an appropriate early intervention is obligatory to support or induce auditory-based speech and language acquisition [using hearing aids (HA) or cochlear implantation (CI)]. If a child with severe to profound SNHL experiences limited or no improvement from auditory rehabilitation using HA, CI should be performed [5].

This current ABR study was aimed at investigating the auditory threshold changes and auditory pathway maturation within the first year of life. The effects of early therapy based on the results of the ABR measurement were evaluated and the reliability of AEPs as a tool for early examination of auditory pathway maturation leading to treatment indications during the first year of life was investigated.

## Materials and methods

**Fig. 1:**
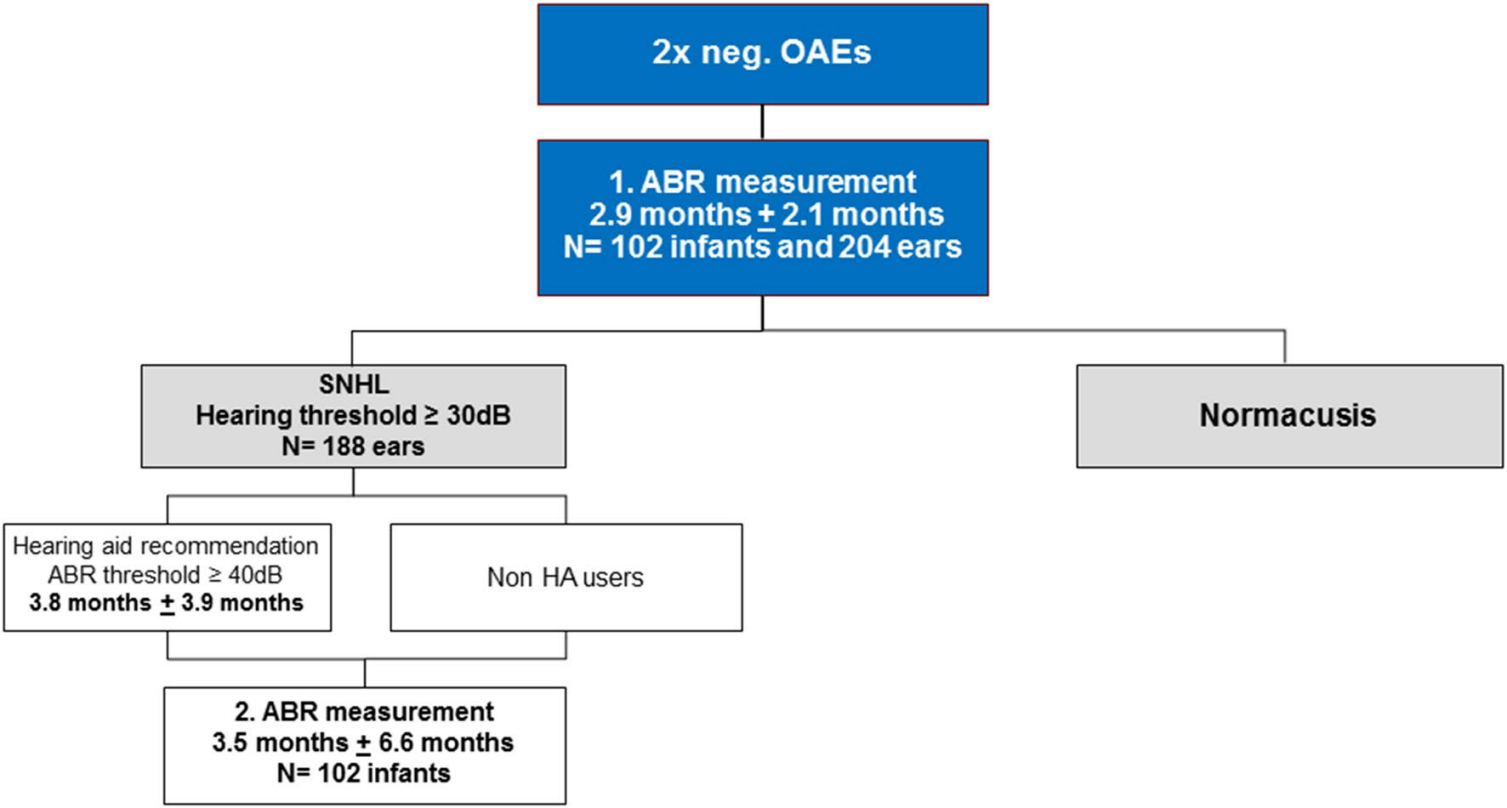
Flowchart of staged additional hearing testing of infant before the first year of life

We retrospectively reviewed the medical records of 102 infants who were referred to a tertiary care hospital between March 2012 and March 2019 and received two or more ABR measurements within the first year of life. The study group consisted of 33 female and 69 male, 18 premature and 84 full-term infants, and 29 infants (28.4%) that were identified to have risk factors that are listed in Table 1. Seventeen infants had unilateral HL and 85 bilateral HL. Eighty infants underwent 2 ABR testing and 22 infants 3 before 12 months of age. The first ABR measurement was performed at a median age of 2.9 months ± 2.1 months and the second at a median age of 3.5 months ± 6.6 months. In the case of a third measurement, it was performed at a median age of 7.1 months ± 1.9 months (Fig. 1).

**Table 1:** Frequency of prenatal, perinatal and postnatal risk factors and comorbidities with possible effect on HL

Prenatal	
Connexin 26	1
Emanuel syndrome	1
Patau syndrome	1
Large vestibular aqueduct syndrome	2
Down syndrome	2
Prematurity	18
Perinatal	
RSV infection	1
Sepsis	2
Low weight	6
Asphyxia	7
Hyperbilirubinemia	13
Postnatal	
Meningitis	1

ABR measurement (Nicolet Synergy EDX, Natus Medical Incorporated, Pleasanton, CA, USA) was performed during postprandial natural sleep. For ABR recordings, a click was used (100 µs, alternating polarity), the stimulation rate was 19.1 Hz, and 2000 averages were used and delivered through a pair of TDH-39P audiometric earphones (Telephonics Corporation, Farmingadale, NY, USA). The latency and amplitude of wave Jewett I, wave Jewett V and IPL I–V were registered and compared to norm values for the age groups (Table 2). Typically with measurements in natural sleep, stimulation was started with 60 dB above normal hearing level (nHL). In case of a suspected asymmetrical hearing, a contralateral masking was used. An experienced audiologist evaluated the recorded potentials.

**Table 2:** Threshold changes between the first and last ABR measurement on 102 right (R) and 102 left (L) ears separated into groups with and without the use of HA

	No change	I of 10dB	I of 20dB	I of 30dB	I of 40dB	I of 60dB	D of 10dB	D of 20dB	D of 30dB	D of 40dB
Mild HL R with HA	3						1	1		
Mild HL R without HA	2	6	7						1	
Moderate HL R with HA	3	9	2	4	2		1			1
Moderate HL R without HA		2	1	6	2					
Severe HL R with HA	1	4	1	1						
Severe HL R without HA							1			
Profound HL R with HA	11		2	1	1					
Profound HL R without HA	18									
Mild HL L with HA		2					3			
Mild HL L without HA	1	5	2				1			1
Moderate HL L with HA	6	9	4	2	1		1		1	1
Moderate HL L without HA	1	2	1	5	2		1	1		
Severe HL L with HA		2								
Severe HL L without HA		1				1				
Profound HL L with HA	12	1			1					
Profound HL L without HA	22									

ABR thresholds at 20 dB nHL or lower were considered as normal, thresholds between 30 and 40 dB nHL were considered as mild HL, thresholds between 50 and 60 dB nHL as moderate HL, thresholds between 70 and 80 dB nHL as severe HL and thresholds higher than 90 dB nHL as profound HL. Seven right ears showed a normacusis, 21 a mild HL, 33 a moderate HL, eight a severe HL and 33 right ears had a profound HL. Nine left ears had a normacusis, 15 a mild HL, 38 a moderate HL, four a severe HL and 36 a profound HL.

Adequate treatment decisions were determined on the basis of the clinical and audiological findings. Conductive HL is the most frequent complication of serous otitis media, typically owing to the increased stiffness and mass of the tympanum caused by middle ear effusion [26]. In the case of suspected middle ear effusion, a conservative treatment and “watchful waiting” were recommended before the follow-up ABR measurement (about 4 to 6 weeks after the initial ABR measurement). The middle ear effusion was diagnosed based on the otoscopic findings by an experienced otolaryngologist and/or an ongoing infection of the upper airway with rhinorrhea during a consultation. Infants with suspected conductive HL due to middle ear effusion were initially not provided with HA. In the case of persistent conductive HL shown in the follow-up ABR measurement, HA were recommended to minimize the effect on decreased auditory input and when indicated/appropriate, myringotomy with placement of pressure equalization tubes. Table 2 shows an overview how diagnoses regarding SNHL, conductive HL or delayed auditory pathway maturation were determined based on the ABR measurement results of wave Jewett I and V and IPL I–V (Table 2).

Recommendations for determining HA use:

- infants with aiding uni- or bilateral HL with diagnostic ABR thresholds of 40 dB nHL and worse (up to 95nHL)
- when anatomically possible (sufficient external ear and canal anatomy to support the coupling of an earmold and retention of the device)
- in case delayed auditory maturation depended on the prolonged latency of IPL I–V

For parents, it is an essential foundation to be provided with complete information to help them resolve barriers to effective daily management and acquire support for skill acquisition. Therefore, they were supported from early intervention programs either home based or center based to learn to navigate challenges with HA fitting of their infants more effectively.

Follow-up ABR testing was conducted within 1 to 3 months to investigate changes in hearing thresholds, review the current therapy, and if necessary indicate further treatment. If a gradual progression of the HL was recognized in the ABR measurements up to the first year of life, we followed all further measurements of the infants.

HA were provided at a median age of 3.4 months ± 2.6 months for the right ear and at a median age of 4.1 months ± 5.2 months for the left ear. The infants were provided with HA at all degrees of HL. In total, 10 ears with mild HL (five right ears and five left ears), 47 with a moderate HL (22 right and 25 left ears), 9 with a severe HL (seven right and two left ears) and 29 ears with a profound HL (15 right ears and 14 left ears) were equipped with HA. In the follow-up consultations, the parents reported that the infants had accepted the use of the HA devices and showed good acceptance.

To analyze the effect of the hearing amplification on the auditory maturation, we looked at two different subgroups of infants with moderate HL. In the majority (68%) of the ABR measurements of the infants with moderate HL, wave Jewett I and wave Jewett V were identified that allowed the assessment of IPL I–V.

The first subgroup was composed of all infants with moderate HL separated into right and left ears and HA users and non-HA users.

In the second subgroup, we further looked at infants with identified risk factors with moderate HL (only those in whom the determination of IPL I–V was possible) provided with HA and separated them from infants fitted with HA without risk factors to identify if risk factors possibly affect auditory maturation.

The data obtained were organized into tables and figures, using descriptive statistics with Microsoft^®^ Office Excel 2010. Results were analyzed and plotted in a custom-written Matlab 2017a (The MathWorks, Inc., Natick, MA, USA). As a criterion of significance, a 95% confidence level (*p* < 0.05) in the Student ‘s *t* test was considered as significant.

Ethic approval is pronounced positively from MHH-Ethics committee.

## Results

Fifty-eight infants (56.9%) showed threshold changes in the follow-up testing on the right ear and 51 infants (50.0%) on the left ear. An improvement was recognized in 50 right ears (49.0%) and 41 left ears (40.2%) and deterioration in eight right ears (7.8%) and ten left ears (9.8%). By how much dB the hearing threshold changed depending on the severity of the HL and treatment with HA between the first and last ABR measurements is summarized in Table 3. Twenty-one right ears (22.1% of 95 infants) and twelve left ears (12.9% of 93 infants) recovered to normal hearing in their last ABR measurement. Out of the 21 right ears, 10 had a mild HL (all without the use of HA) in the first evaluation and 11 a moderate HL (63.6% with HA and 36.4% without HA). From the twelve left ears six had a mild (83.3% without HA, 16.7% with HA), five a moderate (60.0% without HA, 40.0% with HA) and one a severe HL (without HA) in the initial ABR measurement.

**Table 3:** Standard values of wave Jewett I (at 80 dB nHL), wave Jewett V (at 80 dB nHL) and IPL I–V for different months are listed first [11]

	Wave Jewett I at 80dB nHL (ms)	Wave Jewett V at 80dB nHL (ms)	IPL I–V (ms)
3–6 months	1.59	6.25	4.65
6–9 months	1.59	6.10	4.50
9–12 months	1.59	5.91	4.32

	Wave Jewett I	Wave Jewett V	IPL I–V	Interpretation
	First ABR measurement			
1	Prolonged	Highly prolonged	Prolonged	Delayed auditory maturation
2	Highly prolonged	Highly prolonged	Prolonged	Middle ear effusion + delayed auditory maturation
3	Highly prolonged	Highly prolonged	Normal	Middle ear effusion
	Last ABR measurement			
1	Normal	Shortened	Normal	Auditory pathway maturation
2	Shortened	Shortened	Shortened	Cured middle ear + auditory pathway maturation
3	Shortened	Shortened	Normal	Cured middle ear + physiological maturation

Below the interpretation of the results of the first ABR measurement and their corresponding last ABR measurement illustrated on three different cases

*I* Improvement, *D* Deterioration

No hearing threshold change was seen in 43 right ears (42.2%) and 51 left ears (50.0%) in the follow-up ABR testing. Despite no apparent change in thresholds, a reduction of the IPL I–V was seen in the infants with mild-to-moderate HL on both sides. 29 right ears and 34 left ears were diagnosed with a profound HL in the initial ABR measurement and the IPL I–V could not be measured. Twenty-four right ears and 22 left ears with a profound HL at the time of the repeated ABR measurement were provided with CI (at a minimum age of five months). In preparation for a CI surgery, a detailed audiological and radiological diagnostic using computer tomography (CT) and magnetic resonance tomography (MRT) of the temporal bones was carried out under general anesthesia. CI was performed at the age of 9.3 months ± 4.0 months on the right ear and at the age of 10.4 months ± 4.5 months on the left ear.

A progression in HL was recorded in 19 ears (9.3% of 204 ears) (Table 4). Thirteen showed a unilateral and three a bilateral threshold deterioration. Seven of the infants were identified with risk factors and showed a progression up to 40 dB. Table 4 summarizes the risk factors, ABR thresholds, age at measurements and recommended therapy. Up to now, the children are called in at intervals of 6 to 12 months for clinical and audiological examinations using ABR measurements and—depending on the age—pure tone audiometry to control for optimally fitted HA.

**Table 4:** Results in the ABR measurements of infants with progressive hearing loss, diagnosed risk factors and recommended therapy

No.	Risk factor	1. ABR threshold right/left, age	HA provision right/left	2. ABR threshold right/left, age	Last follow-up ABR threshold right/left, age	Recommendation
1	Prematurity, hyperbilirubinemia	40 dB/60dB, 2 months	Yes/yes	50dB/60dB, 4 months	50dB/60dB, 24 months	HA both sides
2	No	60dB/60dB, 3 months	Yes/yes	80dB/60dB, 7 months	70dB/30dB, 19 months	HA both sides
3	No	20dB/60dB, 3 months	No/yes	20dB/70dB, 7 months	20dB/70dB, 18 months	HA left
4	No	60dB/30dB, 1 months	No/no	60dB/50dB, 3 months	20dB/20dB, 12 months	middle ear ventilation surgery
5	No	70dB/20dB, 2 months	Yes/no	80db/20dB, 3 months	50dB/20dB, 14 months	HA right
6	No	40dB/50dB, 3 months	Yes/yes	50dB/80dB, 10 months	60dB/60dB, 24 months	HA both sides
7	No	20db/30dB, 4 months	No/no	40dB/40dB, 10 months	20db/20dB, 12 months	conservative therapy, serous otitis media
8	No	30dB/40dB, 3 months	No/no	40dB/40dB, 5 months	20dB/20dB, 24 months	middle ear ventilation surgery
9	Prematurity, hyperbilirubinemia, patau syndrome	90dB/60dB, 1 months	Yes/yes	90dB/80dB, 5 months	90dB/90dB, 7 months	CI both sides
10	LVAS both sides	95dB/60dB, 0 month	Yes/yes	95dB/80dB, 5 months	95dB/90dB, 19 months	CI both sides
11	LVAS both sides	50dB/50dB, 5 months	Yes/yes	70dB/60dB, 10 months	95dB/95dB, 15 months	CI both sides
12	Dehiscence of the superior semicircular canals	95dB/50dB, 5 months	Yes/yes	95dB/80dB, 5 months	95dB/95dB, 16 months	CI both sides
13	Prematurity, CMV infection, hyperbilirubinemia	20dB/95dB, 1 month	No/yes	40dB/95dB, 3 months	30dB/95dB, 9 months	HA right, CI left
14	No	50dB/50dB, 3 months	Yes/yes	50dB/60dB, 5 months	50dB/60dB, 18months	HA both sides
15	No	40dB/50dB, 8 months	Yes/yes	60dB/50dB, 12 months	60dB/50dB, 27 months	HA both sides
16	Prematurity, hypoxia	50dB/50dB, 3 months	Yes/yes	60dB/50dB, 6 months	60dB/50dB, 24 months	HA both sides

### First subgroup of infants with moderate HL

**Fig. 2:**
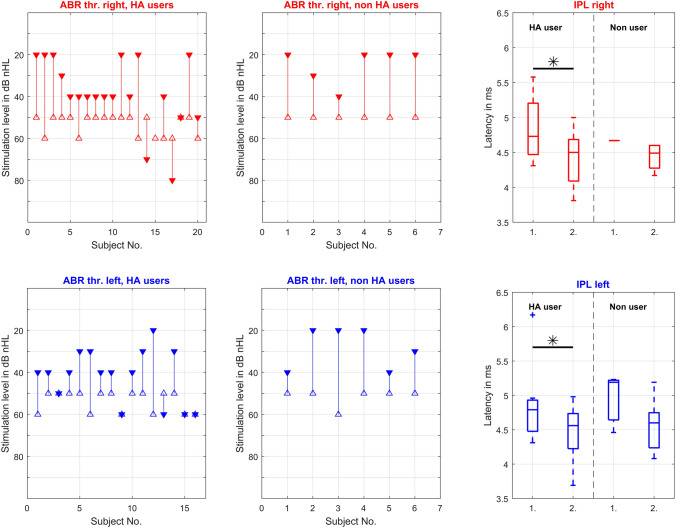
Results of the first subgroup of all infants with moderate HL: Threshold changes between the first △ and last ▼ ABR measurement of the right and left ears equipped with HA and non-HA user. The ★ marked if there were no threshold changes seen between the first and last ABR measurements. The boxplots on the right show the difference between the IPL I–V measured at the first (1) and last (2) ABR evaluation of HA users and non HA users. The outer limits of each box represent the 25th and 75th percentiles, with the median shown as the line within the box. Whiskers indicate the 5th and 95th percentiles and more data points (crosses) displayed the outliers. The star marks the significant differences between the one and two IPL I–V in the group of HA users

The differences between the threshold changes and IPL I–V recorded for each ear diagnosed with moderate HL with and without HA are shown in Fig. 2. In the group of HA users, the IPL I–V changed from average 4.84 ms at the mean age of 3.6 months to average 4.44 ms at the mean age of 8.2 months on the right side and from average 4.92 ms at 3.4 months to average 4.48 ms at 7.3 months on the left side. In the group of infants who were not equipped with HA, the IPL I–V changed from average 4.67 ms at the mean age of 1.2 months to average 4.44 ms at 2.8 months on the right side and from average 4.96 ms at the mean age of 2.7 months to average 4.55 ms at 5.2 months on the left ear. The average IPL I–V reductions of the HA-equipped infants showed statistically significant differences on both sides. The average IPL I–V reductions of the non-HA users were not statistically significant.

### Second subgroup of infants with moderate HL and emphasis on risk factors

**Fig. 3:**
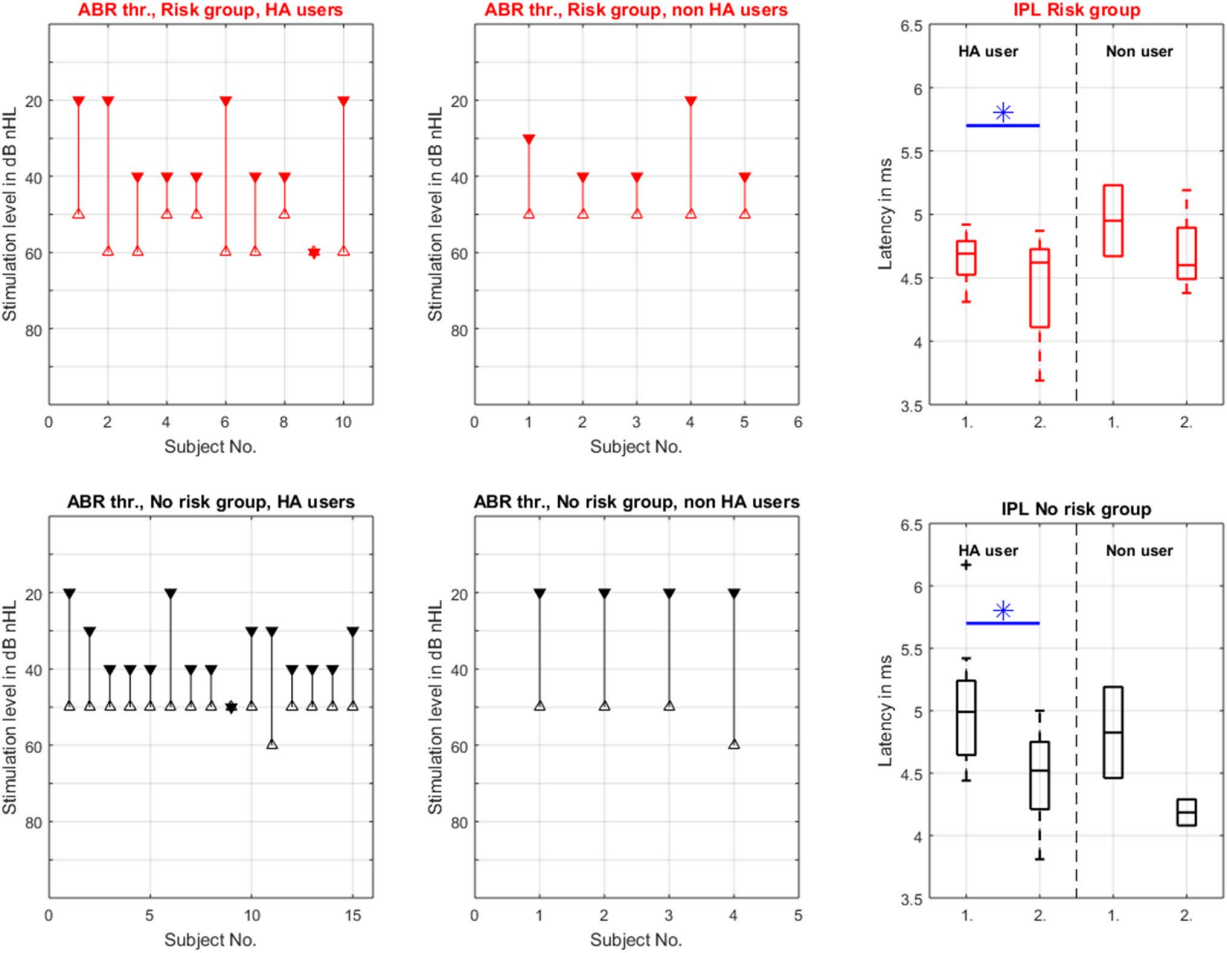
Results of the second subgroup of infants with moderate HL and emphasis on risk factors: Threshold changes between the first △ and last ▼ ABR measurement of the infants with and without risk factors equipped with HA and non HA user. The ★ marked if there were no threshold changes seen between the first and last ABR measurements. The boxplots on the right show the difference between the IPL I–V measured at the first (1) and last (2) ABR evaluation of HA users and non HA users. The outer limits of each box represent the 25th and 75th percentiles, with the median shown as the line within the box. Whiskers indicate the 5th and 95th percentiles and more data points (crosses) displayed the outliers. The star marks the significant differences between the one and two IPL I–V in the group of HA users

In this subgroup analysis, there was no separation between right and left ears; only the distribution of risk factors was accounted for (Fig. 3). In the group of infants with risk factors who received HA, the IPL I–V changed from average 4.66 ms at the mean age of 4.6 months to average 4.42 ms at the mean age of 8.8 months. In the group of infants with risk factors who did not receive HA, the IPL I–V changed from average 4.95 ms at the mean age of 2.2 months to average 4.69 ms at the mean age of 4.6 months. The IPL I–V from infants without risk factors after the use of HA shortened from 5.05 ms at 2.0 months to 4.48 ms at 5.9 months. In infants without risk factors and without HA use, the IPL I–V showed a shortening from 4.79 ms at 2.3 months to 4.22 ms at 4.0 months. The average IPL I–V reductions of the HA equipped infants with and without risk factors showed statistically significant differences. The average IPL I–V reductions of the non-HA users with and without risk factors were not statistically significant.

## Discussion

The NHS leads to early detection of HL and its value consists in the potential early and physiological start of hearing also in hearing impaired infants. In children with a diagnosed SNHL, auditory maturation is delayed and the maturation delay can also be influenced by other risk factors [27–29]. In the present retrospective study, we examined in a population of 102 infants whether the development of hearing postpartum and auditory maturation can be depicted with AEPs at a very early age, i.e. before the first year of life. To our knowledge, this is the largest study including such young patients. We indicated an early therapy intervention and examined the results in the follow-up ABR measurement. Likewise, we investigated the feasibility of quality control in infants after early HA fitting via ABR with the means of examination of the ABR threshold changes and IPL I–V. Although there have been numerous studies evaluating the outcomes of children with HL over the past century, none of them have focused specifically on infants at a very early age with SNHL and their outcome after early intervention. All the infants in our study presented an abnormal ABR result of ≥ 30 dB nHL in at least one ear and received two or more ABR measurements within the first year of life.

In the first stage of NHS, only a potential hearing disorder is detected. The diagnostic confirmation verifies the HL and, ideally, also determines the hearing threshold and location of hearing impairment. The location of hearing impairment differentiates between a conductive HL caused by middle ear effusion and/or an additional SNHL. The most reliable, non-invasive method to evaluate the auditory maturation processes is the ABR. The auditory system can be measured from the cochlear through the auditory nerve into the brainstem [4]. This method is independent of the cooperation, a well-established method for decades and is the most important tool in diagnosing hearing impairment in infants with a repetitively negative NHS result. While ABR thresholds are important in establishing the degree of HL, ABR latencies and potential morphology help differentiate between different types of HL and identifying delayed auditory maturation (Table 2) [10]. In cases of diagnosed HL, there is a possibility of improvement or worsening of the hearing thresholds. To detect threshold changes in infants at an early stage, follow-up ABR measurements at short intervals are required. The necessity of further therapy can then be derived from the course of the measurements and their results. In about 50% of the cases in our study, an improvement of hearing threshold was seen. Many previous studies evaluated changes in hearing status of infants with congenital HL. They drew attention to the need of clear discrimination of type of HL being a fundamental component of auditory rehabilitation and treatment decision-making [24, 25, 30, 31].

It is known that the age at HA fitting is an important factor on the outcomes of children with HL [32]. Based on the result of the initial ABR, HA were immediately prescribed as mentioned above. This implies that the youngest infants received their HA within the first month of life. Retrospectively our recommended early intervention was accepted in all cases, the prescribed HA showed good acceptance and the devices were worn consistently. This is remarkable because the retrospective study results show for the first time in the literature and on the largest number of 102 included infants to date that this clinical procedure is feasible in clinical daily routine, outside a clinical study procedure.

In accordance with the literature, we demonstrate in this study group a diagnostic proof of maturation of the auditory pathway up the brainstem by the IPL I–V, especially in our subgroups of infants with moderate HL. We compared the results of the ABR measurements of two subgroups of infants with or without risk factors with moderate HL after the use of HA versus non-HA users. The significant shortening of IPL I–V in all groups (with and without risk factors) who received HA assumed auditory pathway maturation (Figs 2, 3). Our results also suggest that infants with risk factors as described in the literature showcase a delayed auditory pathway maturation compared to infants without risk factors, independent from hearing amplification. Confounding factors that need to be taken into account are the different ages of the non-HA users in both subgroups at the first measurement as well as different time intervals for follow-up examinations.

In a study of Moreno-Aquirre et al. with 13 infants with perinatal brain injury and severe to profound HL, threshold changes were also determined after the early use of HA on the right ear with the left ear being the control side before the age of 6 months [2]. In their study in nine HA right ears and nine non-HA left ears, the IPL I–V could be determined. The IPL changes in the follow-up measurements were significant in the right ears and not significant in the control left ears. These results were also evident in our group of HA users and non-HA users of both subgroups. An early intervention using optimally fitted HA showed a high impact on the auditory rehabilitation and our results indicate an improvement of the hearing thresholds within the first 12 months of life.

Giving the retrospective nature of this study, we can only speculate whether these improvements of hearing levels may have occurred without an early use of HA. Evaluating the effect of hearing amplification would need a larger prospective case–control study, but such a study would encounter other ethical challenges pertaining to treatment delivery.

In the groups of non-HA users, we investigated decreasing latencies of Jewett V and major improvements of thresholds (in most cases 30–40 dB). Both facts indicate that middle ear effusion might have been the reason for the delayed wave Jewett V and the hearing threshold at the time of the first evaluation. Considering the incidence of middle ear effusions in infancy under the age of 2 years is estimated to be as high as 61% [10, 24, 33]. Since the infants with middle ear effusions had to be controlled for checking and adjusting therapy, these infants were called in at shorter intervals, which is reflected in the time intervals of the follow-up measurements of the non-HA users.

The incidence of progressive HL in children has been reported with an extremely wide range of 4% to 30% in the literature [27]. A study from Barreira-Nielsen et al. [34] analyzed all follow-up audiologic testing from infants who failed the NHS between 2003 and 2013 in Canada. Out of 330 children with detailed audiologic records, 158 children (47.9%) showed deterioration (at least ≥ 10 dB). They also recorded that within 4 years after diagnosing progressive HL in children, the mean decrease was 25.9 dB (average ± 16.4 dB) in the right ear and 28.3 dB (average ± 12.9 dB) in the left ear [34]. In our study, overall 19 ears showed a deterioration of 10 dB to 40 dB in the follow-up ABR measurement (Table 4). By the time of the diagnosed progression, close follow-up ABR measurements were conducted to determine the impairment and timely initiate an adequate further therapy. In all infants with threshold deteriorations of more than 20 dB, a correlation between influencing factors for HL such as risk factors and comorbidities were seen (Table 4). This implies that in particular infants with risk factors need to be examined more closely after failing the NHS.

Furthermore, in case of a progressive HL, an early diagnosis achieved by appropriate diagnostic investigations including MRI and CT imaging and detailed genetic analyses is necessary. In this study, CT imaging showcased the etiology of progressive HL in three cases (LVAS and dehiscence of the superior semicircular canals). In three cases of progressive HL, the infants showed normalization in hearing in the follow-up ABR measurements after the first year of life. In another six cases, the hearing threshold remained stable after the first impairment and the children showed a good acceptance of the HA.

Taking all the cases into account that showed a change of the hearing threshold in this study—improvement or deterioration—we underline the importance of auditory monitoring of infants using AEPs before the first year of life. Identifying the hearing impairment within the first months of age and starting with HA as first-line therapy leads also to an adequate diagnostic procedure and threshold confirmation towards the indication for CI. The confirmation of the hearing level at an age of 6 months helped the infants in our study to receive CI after auditory stimulation at a median age of 9.8 months for the right ear and 11.5 months for the left ear. Lammers et al. reported in 2015 that the median age at implantation decreased significantly because of effectively performed NHS. Over the period from 1995 to 2001, in the center UMC Utrecht and our ENT center, it decreased from 3.4 years to 0.9 years, and 3.1 years to 1.9 years, respectively [35]. This study reveals another drastic decrease of the median age of implantation from 1.9 years in 2001 to 10.7 months in 2019 within this tertiary hospital.

The results of this study also have implications for introducing national NHS tracking in Germany as a consequence of the first part of NHS. This tracking is not only responsible for the quality of identifying the potential hearing-impaired subjects within the first screening 4 days after birth. The second important goal is to ensure the diagnostic procedure and the initiation of the HA therapy. In some countries such as Netherlands, USA and Oman, a tracking system is organized by the insurance companies or the government. Children with a repeated negative hearing screening are referred to an audiology clinic by the screening system. The duties of the NHS tracking are to recommend confirmation of diagnosis and monitor whether it is performed and if therapeutic action takes place. Results from the national tracking system in Netherlands showed that in 2011, 50% of the children with suspected hearing impairment were referred within the desired standard of 24 days [35].

In conclusion, auditory function in infants can improve after early acoustic stimulation with HA, paralleled by an increase in the hearing threshold based on ABR measurements. Furthermore, the auditory maturation can be detected at the age of 6 months or even earlier. The ABR results support diagnosis, timely initiation of therapy and therapy adjustments. The staged additional hearing testing of infants is essential to monitor the development of the hearing level at a very early age using repeated ABR measurements (Fig. 1) not only to identify hearing impairment but also to detect any threshold changes which are relevant for therapeutic decision-making. This might include final treatment strategy decisions regarding surgical procedures such as CI.
